# The ecological footprint of healthcare: awareness, knowledge, and attitudes of medical students and medical residents

**DOI:** 10.3389/fpubh.2025.1711363

**Published:** 2025-11-05

**Authors:** Adele Sarcone, Silvia Angelillo, Gianfranco Di Gennaro, Maria Grazia Belfiore, Claudia Pileggi, Davide Costa, Carmelo Giuseppe Angelo Nobile

**Affiliations:** ^1^Department of Health Sciences, School of Medicine, University “Magna Gracia” of Catanzaro, Catanzaro, Italy; ^2^Department of Psychology and Health Sciences, Pegaso University, Naples, Italy; ^3^Department of Medical and Surgical Sciences, University “Magna Gracia” of Catanzaro, Catanzaro, Italy; ^4^Department of Experimental and Clinical Medicine, School of Medicine, University “Magna Gracia” of Catanzaro, Catanzaro, Italy; ^5^Department of Pharmacy, Health and Nutritional Sciences, University of Calabria, Cosenza, Italy

**Keywords:** ecological footprint, healthcare sustainability, climate change, medical education, environmental health

## Abstract

**Introduction:**

Climate change is widely recognized as the greatest global health threat of the twenty-first century, yet the healthcare sector paradoxically contributes around 4–5% of global greenhouse gas emissions. Although health professionals are well positioned to mitigate this impact, sustainability education remains insufficient in medical curricula. This study aimed to assess the awareness, knowledge, and attitudes of medical students and residents in Italy regarding healthcare’s ecological footprint, with the goal of identifying gaps in training and informing curricular development.

**Methods:**

A cross-sectional survey was conducted between February and June 2024 at the University of Magna Graecia of Catanzaro. A structured, self-administered questionnaire collected socio-demographic data, knowledge of sustainability concepts, concerns about pollution and climate change, attitudes toward education, preferences for single-use versus reusable medical devices, proposed sustainability measures, and perceived barriers.

**Results:**

Logistic regression was used to identify predictors of high concern about pollution and climate change, while Poisson regression examined factors influencing a composite knowledge score. In total, 638 participants were enrolled (mean age 26.8 years; 66% female). Concern was high, with 95% reporting worry about the health impacts of climate change and 73% expressing concern about healthcare-related pollution. Knowledge levels varied: while familiarity with the greenhouse effect was nearly universal, 12% had never heard of the ecological footprint and 28% reported self-directed study. Most participants (84%) supported integrating sustainability into both classroom and clinical training, and 89% recognized the responsibility of healthcare professionals to reduce pollution. Multivariable analysis showed that female gender, older age, and enrollment in nursing or residency programs predicted higher concern, while greater climate change concern, valuing sustainability in medical device use, and support for curricular integration were associated with higher knowledge scores.

**Discussion:**

Overall, Italian medical students and residents show strong engagement with environmental health, but significant knowledge gaps persist. Integrating structured climate-health and sustainability education into medical training is essential to prepare future healthcare professionals to lead environmentally sustainable practice.

## 1 Introduction

Climate change represents the most significant global health threat of the 21st century, with the World Health Organization (WHO) estimating hundreds of thousands of premature deaths annually due to climate-related diseases such as malnutrition, vector-borne infections, and heat stress (1). The healthcare sector, while tasked with protecting health, paradoxically contributes substantially to climate change, responsible for approximately 4–5% of global greenhouse gas emissions (2, 3). This contribution stems from energy-intensive infrastructure, procurement, and waste generation, with emissions projected to rise unless urgent systemic changes occur (2).

The healthcare systems accounts for around 4% of greenhouse gas emissions, emphasizing the importance of strategies to reduce environmental impact (4). Globally, the largest healthcare carbon footprints are found in the United States, China, and the European Union, which together represent over half of the sector’s emissions (3).

In response, the Lancet Commission framed climate change not only as a threat but as the greatest global health opportunity, urging health professionals to take leadership in climate mitigation and adaptation efforts (5). Healthcare professionals are uniquely positioned to influence change by promoting sustainable clinical practices, such as the use of low-carbon medical devices, waste reduction, and telemedicine to minimize patient and staff travel (2). Despite this pivotal role, education on healthcare sustainability remains insufficient.

Ryan et al. surveyed medical, nursing, and physician assistant students in the United States and found that while students demonstrated strong concern for climate change and pollution, many reported inadequate training on how to address these issues in clinical practice (6). Barriers included lack of curriculum content, time constraints, and limited awareness of healthcare’s ecological footprint. Research shows that while physicians recognize the importance of climate change and its health effects, they feel insufficiently prepared due to gaps in professional education. Medical students, including those in China (7) and the United States (US) (8), understand the health impacts of climate change but often lack confidence and formal training to address them effectively. Studies consistently support the integration of climate change and health topics into medical education. Although core educational goals have been developed, there is currently no mandatory requirement for health professionals to receive this training.

This educational gap has been acknowledged by the WHO, which highlights the need for climate-resilient and environmentally sustainable health systems, including training healthcare workers to build capacity for sustainable practice and resilience against climate impacts (1). Integrating environmental sustainability into health professional curricula is thus critical to empower future clinicians to reduce healthcare’s ecological footprint effectively.

Building on these international insights, this study investigates the awareness, knowledge, and attitudes regarding healthcare’s ecological footprint among medical students, health professionals students, and specialist trainees at the University of Magna Graecia in Italy. Understanding the current educational needs and perceptions is essential for informing curricular innovations that prepare health professionals to lead sustainability efforts within healthcare systems.

## 2 Methods

### 2.1 Study design, participants, and sampling procedures

A cross-sectional study was carried out between February 2024 and June 2024 and included students aged 18 years or older enrolled at the Magna Graecia University of Catanzaro.

The research team approached students on randomly selected days during their courses to invite them to participate in the study. Data were collected using a self-administered questionnaire, which took approximately 15 min to complete. At the start of the questionnaire, students were provided with information outlining the main objectives of the survey. They were informed that participation was entirely voluntary and anonymous, that their responses would be treated with strict confidentiality, and that they could withdraw from the survey at any point during completion without any consequences. It was also clarified that there would be no financial compensation for participation and that the data would be used solely for the stated research purposes.

The questionnaire collected information across seven main areas:

1. Socio-demographic and academic characteristics, including gender, age, nationality, region of residence, degree program, and specialization.
2. Knowledge of sustainability and healthcare’s ecological footprint, assessed through multiple-choice questions on concepts such as the Sustainable Development Goals, greenhouse effect, and resilience.
3. Perceived concern about pollution and climate change, measured with two items on a four-point Likert scale ranging from “not at all” to “very much.”
4. Attitudes toward education and training, exploring opinions on the inclusion of environmental sustainability and pollution-related topics in medical curricula, using five-point Likert scales from “strongly disagree” to “strongly agree.”
5. Preferences regarding single-use and reusable medical devices, used as an applied example of how clinical choices can affect the ecological footprint of healthcare. This section explored factors influencing the choice of device type—such as infection control, cost, environmental impact, and availability—and asked respondents to rate their agreement with statements about safety and sustainability using five-point Likert scales. This part of the questionnaire was intended to illustrate how adopting reusable medical devices, when clinically appropriate, can contribute to reducing the ecological footprint in the healthcare sector.
6. Proposals to reduce pollution in healthcare, including agreement with strategies such as telemedicine, transparency in suppliers’ ecological footprints, and staff training.
7. Perceived barriers to promoting sustainability, such as lack of training, workload, or misconceptions about reusable equipment.

Items were primarily measured on five-point Likert scales, allowing respondents to express levels of agreement or concern. A pilot test involving 50 health sciences students confirmed the clarity and internal consistency of the instrument, leading to minor revisions before its final use.

Prior to distributing the questionnaire, a pilot study was carried out with a small sample of 50 students from the health sciences field to verify the clarity and comprehensibility of the questions and to ensure they could be answered with ease.

The study protocol was approved by the Territorial Ethics Committee of the Calabria Region (Protocol n.20, 30/01/2024) and was conducted in accordance with the Declaration of Helsinki. All participants gave informed consent to participate after having been given full informations about the study.

### 2.2 Statistical analysis

Continuous variables were summarized as means and standard deviations when normally distributed, or as medians and interquartile ranges in the presence of skewed distributions.

The study assessed two main outcomes, consistent with the study objectives described in the abstract. The primary outcome was the overall level of concern about environmental and health impacts, defined by combining responses on concern about pollution generated by the healthcare sector and concern about the health impacts of climate change. Participants reporting moderate or high concern for both items were coded as 1 (high concern), and all others as 0 (low or no concern).

The secondary outcome was a composite knowledge score, reflecting the participants’ understanding of key sustainability and health concepts, including the Sustainable Development Goals and Agenda 2030, the ecological footprint, greenhouse effect, resilience, social gradient in health, health inequalities, and determinants of health. One point was assigned for each concept known (“I’ve heard of it,” “I studied it at school,” “I studied it at university,” or “I did my own research”), while no points were assigned to “I’ve never heard of it.” Higher total scores therefore indicate greater knowledge of sustainability in healthcare.

For the primary outcome, stepwise logistic regression was used to identify predictors of high concern about healthcare-related pollution and climate change. The independent variables included age, sex, and type of academic program (medicine, nursing, residency, or other). Additional predictors captured prior knowledge of sustainability concepts and attitudinal responses from three questionnaire domains:

- opinions on education and training;
- factors influencing the choice between single-use and reusable medical devices;
- perceived barriers to promoting sustainability in healthcare.

All Likert-scale variables were treated as approximately continuous. For the secondary outcome, a Poisson regression model was applied to examine factors associated with higher knowledge scores. Independent variables included socio-demographic characteristics, level of concern about pollution and climate change, and attitudinal responses from Sections D, E, and G. This model therefore complements the primary analysis by identifying factors linked with greater understanding of sustainability-related concepts.

Both models were built using backward variable selection with a removal criterion of *p* > 0.20, and statistical significance was set at *p* < 0.05. Model fit was evaluated using the Hosmer–Lemeshow goodness-of-fit test, and all analyses were performed using Stata version 19 (StataCorp, College Station, TX, United States).

## 3 Results

The results are presented following the structure of the Methods section. First, the main socio-demographic characteristics of participants are described. Subsequently, findings on knowledge, attitudes, and practices related to sustainability and environmental awareness are reported, followed by responses regarding proposed actions and perceived barriers. Levels of concern about healthcare-related pollution and climate change are then presented, and finally, the multivariable analyses explore factors associated with high concern and higher knowledge scores.

### 3.1 Participant characteristics

A total of 638 participants were included in the study (Table 1). The mean age was 26.77 ± 6.52 years, and 65.99% (*n* = 421) were female. Nearly half were enrolled in the medicine and surgery program (46.39%; *n* = 296), followed by nursing (23.98%; *n* = 153), residency programs (19.59%; *n* = 125), and other health-related programs (10.03%; *n* = 64). Among residents, 27.2% (*n* = 34) were in medical specialties, 27.2% (*n* = 34) in surgical specialties, and 45.6% (*n* = 57) in clinical services.

**Table 1:** Main characteristic of the respondents.

Characteristic	*N*	%	Mean ± SD
Gender			
Male	217	34.01%	
Female	421	65.99%	
Age			26.77+/6.52
Degree programs			
Medicine and surgery program	296	46.39%	
Nursing program	153	23.98%	
Residency programs	125	19.59%	
Other health-related programs	64	10.03%	
Residency programs area			
Medical area	34	27.20%	
Surgical area	34	27.20%	
Clinical services area	57	45.60%	

### 3.2 Knowledge and educational attitudes

As reported in Table 2, participants showed heterogeneous knowledge regarding sustainability and environmental health concepts. The greenhouse effect was almost universally known, with only 0.63% reporting they had never heard of it. In contrast, 12.23% had never heard of the ecological footprint and 31.19% were unfamiliar with the social gradient in health. Nearly half (47.02%) correctly identified that the Italian healthcare sector contributes about 4% of national greenhouse gas emissions.

**Table 2:** Knowledge about sustainable development and the ecological footprint of healthcare systems.

	I’ve never heard of it *N* (%)	I’ve heard of it *N* (%)	I studied it at school *N* (%)	I studied it during my university studies *N* (%)	I informed myself independently *N* (%)
Sustainable Development Goals (SDGs) 2030 Agenda	172 (26.96)	281 (44.04)	68 (10.66)	9 (1.41)	108 (16.93)
Ecological footprint	78 (12.23)	261 (40.91)	112 (17.55)	11 (1.72)	176 (27.59)
Greenhouse effect	4 (0.63)	90 (14.11)	420 (65.83)	10 (1.57)	114 (17.87)
Resilience	74 (11.60)	253 (39.66)	96 (15.05)	36 (5.64)	179 (28.06)
Social gradient in health	199 (31.19)	195 (30.56)	58 (9.09)	103 (16.14)	83 (13.01)
Health inequalities	55 (8.62)	161 (25.24)	97 (15.20)	167 (26.18)	158 (24.76)
Determinants of health	74 (11.60)	139 (21.79)	64 (10.03)	267 (41.85)	94 (14.73)
Opinions					
	**Strongly disagree**	**Disagree**	**Neither agree nor disagree**	**Agree**	**Strongly agree**
D.1.1. It is important to be aware of these topics, but I do not consider them relevant to patient care	152 (23.82)	243 (38.09)	86(13.48)	92 (14.42)	65 (10.19)
D.1.2. I believe I can learn more about these topics through clinical practice rather than theoretical classroom lessons	46 (7.21)	170 (26.65)	188(29.47)	148 (23.20)	86 (13.48)
D.1.3. I believe these topics should be addressed in the classroom and then further explored during clinical practice	17 (2.66)	21 (3.29)	62 (9.72)	318 (49.84)	220 (34.48)
D.1.4. The workload required by the degree program/specialty training is already too heavy. There is no time to address these topics	86 (13.48)	218 (34.17)	171 (26.80)	95 (14.89)	68 (10.66)
D.1.5. It is important to understand these topics, as healthcare professionals play a crucial role in educating patients about the impact of pollution and climate change on health	26 (4.08)	29 (4.55)	64 (10.03)	234 (36.68)	285 (44.67)
D.1.6. Healthcare professionals have a responsibility to use resources rationally and prevent pollution in their professional practice	16 (2.51)	15 (2.35)	39 (6.11)	229 (35.89)	339 (53.13)
D.1.7. Pollution and climate change are not topics that should be included in degree programs or specialty training	201(31.50)	210(32.92)	92 (14.42)	60 (9.40)	75 (11.76)
D.1.8. I am not interested in these topics	348 (54.55)	178 (27.90)	48 (7.52)	36 (5.64)	28 (4.39)

Most participants supported the inclusion of sustainability topics in medical education: 84.32% agreed these subjects should be taught both in the classroom and reinforced during clinical practice, 81.35% emphasized the educational role of healthcare professionals toward patients, and 89.02% recognized pollution prevention as part of professional responsibility. Only 10.03% expressed disinterest or viewed these topics as irrelevant to patient care.

### 3.3 Clinical practices and ecological awareness

Preferences regarding single-use versus reusable medical devices are summarized in Table 3. About 27.27% preferred reusable devices, 27.74% favored single-use devices, and 22.57% had no preference. Among the factors influencing this choice, 51.41% cited infection control as highly important, 37.93% mentioned availability in the department, 35.89% considered cost moderately relevant, and environmental sustainability was rated as very influential by 28.06% and moderately influential by 29.62% of respondents. In addition, 70.38% agreed that single-use devices reduce the risk of infection.

**Table 3:** Preferences regarding the use of single-use versus reusable medical devices.

	1	2	3	4	5
Habit	126 (19.75%)	103 (16.14%)	209 (32.76%)	90 (14.11%)	110 (17.24%)
Availability in the ward	242 (37.93%)	128 (20.06%)	109 (17.08%)	75 (11.76%)	84 (13.17%)
Infection control	328 (51.41%)	71 (11.13%)	97 (15.20%)	37 (5.80%)	105 (16.46%)
Concern about cost	111 (17.40%)	128 (20.06%)	229 (35.89%)	70 (10.97%)	100 (15.67%)
Environmental sustainability	179 (28.06%)	116 (18.18%)	189 (29.62%)	67 (10.50%)	87 (13.64%)
	**Strongly disagree**	**Disagree**	**Neither agree nor disagree**	**Agree**	**Strongly agree**
There is strong evidence that the use of single-use medical devices significantly reduces the risk of infection	10 (1.57%)	42 (6.58%)	137 (21.47%)	243 (38.09%)	206 (32.29%)

This part of the questionnaire illustrated how clinical decision-making—such as choosing reusable medical devices when clinically appropriate—can directly contribute to reducing the ecological footprint of healthcare.

### 3.4 Proposals and perceived barriers

Respondents strongly supported multiple measures to enhance sustainability (Table 4). 60.34% strongly agreed that industries should provide transparent data on the ecological footprint of supplies and services, 55.49% supported evidence-based recommendations to minimize unnecessary procedures, and 46.24% favored telemedicine for chronic disease management. Nearly half (47.02%) supported investment in leadership and staff training on climate change preparedness, while 42.79% strongly disagreed with the statement that pollution prevention is not the physician’s responsibility.

**Table 4:** Proposals to reduce pollution and promote sustainability within the healthcare sector.

	Disagree	Strongly disagree	Neither agree nor disagree	Strongly agree	Agree
Transparency from industries regarding the ecological footprint of supplies, procedures, and services	9 (1.41%)	18 (2.82%)	34 (5.33%)	385 (60.34%)	192 (30.09%)
Evidence-based recommendations to minimize unnecessary procedures and services	2 (0.31%)	14 (2.19%)	36 (5.64%)	354 (55.49%)	232 (36.36%)
Telemedicine services for patients with chronic diseases	18 (2.82%)	18 (2.82%)	80 (12.54%)	295 (46.24%)	227 (35.58%)
Investment in leadership development and healthcare staff training in climate change prevention and preparedness	12 (1.88%)	6 (0.94%)	61 (9.56%)	300 (47.02%)	259 (40.60%)
The rational use of resources and pollution prevention are not the responsibility of physicians and healthcare professionals	177 (27.74%)	273 (42.79%)	99 (15.52%)	39 (6.11%)	50 (7.84%)
	**Strongly agree**	**Agree**	**Neither agree nor disagree**	**Disagree**	**Strongly disagree**
Transparency from industries regarding the ecological footprint of supplies, procedures, and services	385 (60.34%)	192 (30.09%)	34 (5.33%)	9 (1.41%)	18 (2.82%)
Evidence-based recommendations to minimize unnecessary procedures and services	354 (55.49%)	232 (36.36%)	36 (5.64%)	2 (0.31%)	14 (2.19%)
Telemedicine services for patients with chronic diseases	295 (46.24%)	227 (35.58%)	80 (12.54%)	18 (2.82%)	18 (2.82%)
Investment in leadership development and healthcare staff training in climate change prevention and preparedness	300 (47.02%)	259 (40.60%)	61 (9.56%)	12 (1.88%)	6 (0.94%)
The rational use of resources and pollution prevention are not the responsibility of physicians and healthcare professionals	39 (6.11%)	50 (7.84%)	99 (15.52%)	177 (27.74%)	273 (42.79%)

Main perceived barriers (Table 5) included lack of training about the healthcare sector’s ecological footprint (48.90%), excessive workload (45.77%), and the belief that single-use devices are safer than reusable ones (41.85%). About 25.55% of respondents agreed that rational resource use and pollution prevention are not widely considered priorities among healthcare personnel.

**Table 5:** Potential barriers that may prevent healthcare professionals from promoting sustainability in clinical practice.

	Disagree	Strongly disagree	Neither agree nor disagree	Strongly agree	Agree
Lack of training on the ecological footprint of healthcare systems and the effects of pollution and climate change on health	15 (2.35%)	7 (1.10%)	67 (10.50%)	237 (37.15%)	312 (48.90%)
Excessive workload leading to inefficient use of resources	40 (6.27%)	15 (2.35%)	131 (20.53%)	160 (25.08%)	292 (45.77%)
Belief that single-use devices are safer than reusable ones in preventing infections	53 (8.31%)	16 (2.51%)	90 (14.11%)	212 (33.23%)	267 (41.85%)
	**Strongly agree**	**Agree**	**Neither agree nor disagree**	**Disagree**	**Strongly disagree**
Lack of training on the ecological footprint of healthcare systems and the effects of pollution and climate change on health	237 (37.15%)	312 (48.90%)	67 (10.50%)	15 (2.35%)	7 (1.10%)
Excessive workload leading to inefficient use of resources	160 (25.08%)	292 (45.77%)	131 (20.53%)	40 (6.27%)	15 (2.35%)
Belief that single-use devices are safer than reusable ones in preventing infections	212 (33.23%)	267 (41.85%)	90 (14.11%)	53 (8.31%)	16 (2.51%)

### 3.5 Concern about pollution and climate change

Consistent with the primary outcome, most participants expressed at least moderate concern about both healthcare-related pollution and climate change (Table 5). Specifically, 72.73% of respondents reported moderate or high concern about pollution generated by the healthcare sector, while 94.98% expressed concern about the health impacts of climate change. These findings indicate a strong awareness of environmental threats among respondents and provide the basis for the multivariable analysis of predictors of high concern presented in Table 6.

**Table 6:** Stepwise logistic regression model for the primary outcome (high concern about the health impact of pollution and climate change), using a removal threshold of *p* > 0.20.

Variable	Odds ratio	CI lower	CI upper	*P*
Age	1.06	1.01	1.11	0.017
Gender (female vs. male)	3.08	2.00	4.74	<0.001
Nursing vs. medicine	3.27	1.88	5.69	<0.001
Residency vs. medicine	8.61	3.04	24.34	<0.001
Other health programs vs. medicine	2.60	1.40	4.83	0.003
Knowledge of SDGs (yes vs. no)	1.59	0.99	2.54	0.055
Belief: single-use devices safer	0.75	0.61	0.93	0.009
Concern for cost	1.34	0.99	1.82	0.054
Opinion: not relevant to patient care	0.77	0.63	0.95	0.012
Opinion: better learned in clinical practice	1.59	1.28	1.97	<0.001
Opinion: first in classroom then practice	1.57	1.24	2.00	<0.001
Habit	0.72	0.49	1.05	0.086
Environmental sustainability	1.17	0.95	1.45	0.148
Availability in ward	1.46	0.94	2.26	0.095
Opinion: workload too heavy	1.20	0.96	1.49	0.103
Opinion: not interested	0.65	0.50	0.85	0.001

### 3.6 Predictors of concern and knowledge

In the logistic regression model (Table 6), several variables were associated with higher odds of reporting high concern. Increasing age (OR = 1.06; 95% CI 1.01–1.11) and female gender (OR = 3.08; 95% CI 2.00–4.74) were significant predictors. Compared with medical students, nursing students (OR = 3.27; 95% CI 1.88–5.69), residents (OR = 8.61; 95% CI 3.04–24.34), and students in other health programs (OR = 2.60; 95% CI 1.40–4.83) all showed greater concern. Viewing sustainability topics as irrelevant to patient care was negatively associated with concern (OR = 0.77; 95% CI 0.63–0.95), whereas endorsing their integration into both classroom and clinical education increased the likelihood of high concern (OR = 1.57; 95% CI 1.24–2.00). Students declaring no personal interest (OR = 0.65; 95% CI 0.50–0.85) and those believing that single-use devices are safer (OR = 0.75; 95% CI 0.61–0.93) were less likely to express concern. The model showed good explanatory capacity (pseudo R^2^ = 0.22).

In the Poisson regression model for the knowledge score (Table 7), higher scores were observed among nursing students compared with medical students (β = 0.07; 95% CI 0.00–0.15; *p* = 0.050). Greater importance attributed to environmental sustainability in device choice was positively associated with knowledge (β = 0.07; 95% CI 0.03–0.12; *p* = 0.003), while prioritizing cost showed a negative association (β = −0.06; 95% CI –0.11 to −0.01; *p* = 0.018). Students who believed sustainability topics should first be introduced in the classroom and then reinforced during clinical practice demonstrated higher knowledge scores (β = 0.05; 95% CI 0.01–0.09; *p* = 0.020). Finally, greater concern about the health impacts of climate change was significantly associated with higher knowledge (β = 0.09; 95% CI 0.03–0.14; *p* = 0.002). Although the model explained a modest proportion of variability (pseudo R^2^ = 0.015), associations were consistent across attitudinal and perceptual dimensions.

**Table 7:** Stepwise Poisson regression model for the secondary outcome (knowledge score), using a removal threshold of *p* > 0.20.

Variable	Coefficient	CI lower	CI upper	*P*
Concern for cost	−0.06	−0.11	−0.01	0.018
Availability in ward	0.07	0.03	0.12	0.003
Nursing vs. medicine	0.07	0.00	0.15	0.05
Opinion: first in classroom then practice	0.05	0.01	0.09	0.02
Opinion: better learned in clinical practice	0.03	0.00	0.05	0.066
Concern about the health impact of climate change	0.09	0.03	0.14	0.002

## 4 Discussion

This study explored the level of awareness, knowledge, and attitudes concerning the ecological footprint of healthcare among medical, nursing, and other health-related students, as well as residents in Italy. The sample included 153 nursing students (23.98%), 296 medical students (46.39%), 125 residents (19.59%), and 64 students enrolled in other health-related programs (10.03%). By including participants across different educational stages and disciplines, the study captured diverse perspectives on sustainability within healthcare training. Through two multivariable models, we identified key demographic, educational, and attitudinal predictors associated with higher knowledge and more favorable attitudes toward healthcare sustainability. These findings shed light on the critical role of education and perception in shaping future healthcare professionals’ engagement with environmental issues in clinical settings.

Older age was associated with higher levels of knowledge, possibly reflecting cumulative academic exposure or greater maturity in integrating global health concerns into professional identity. This finding is consistent with previous studies showing that senior students are more likely to perceive climate change as a pressing health concern and engage with its implications in practice (9, 10). Gender was a strong and consistent predictor across both models, with women showing significantly higher odds of sustainability knowledge and pro-environmental attitudes. Prior studies have demonstrated that female healthcare students often express greater concern about environmental health, stronger climate-related risk perception, and greater readiness to act (11, 12). This pattern reflects broader gender differences in environmental values and should be considered when designing targeted educational interventions. Program type also emerged as an important factor: students enrolled in nursing and residency programs demonstrated higher levels of concern compared with medical students, confirming that discipline-specific exposure influences environmental awareness (13, 14). Students in these programs may encounter climate-sensitive health issues more directly, reinforcing the relevance of ecological considerations in clinical care. Moreover, students who recognized core environmental health concepts—such as the greenhouse effect and determinants of health—were more likely to demonstrate informed perspectives, suggesting that scientific literacy is foundational to environmental competence in healthcare (15, 16).

Beyond descriptive analyses, the study applied two multivariable models that provided complementary insights into the determinants of concern and knowledge. The logistic regression model demonstrated that demographic factors, particularly age, gender, and type of program, were strongly associated with concern about the health impacts of pollution and climate change. For example, female participants and those enrolled in nursing or residency programs showed markedly higher odds of concern compared with medical students, highlighting important subgroup differences in environmental awareness. Attitudinal variables also played a central role: students who endorsed the integration of sustainability topics into clinical training or who saw direct clinical relevance were significantly more likely to express concern, whereas perceiving these issues as marginal to patient care reduced the likelihood of concern. These findings underscore the interplay between personal background and curricular framing in shaping climate-related health risk perception (17).

In contrast, the Poisson regression model on the knowledge score highlighted different dynamics. While demographic differences were less pronounced, attitudes toward learning approaches and values related to sustainability emerged as key predictors. Students who emphasized environmental sustainability when considering medical device use or who supported introducing topics first in the classroom and then reinforcing them in practice demonstrated significantly higher knowledge scores. Moreover, concern about climate change was itself a strong predictor of knowledge, suggesting a reciprocal relationship between awareness and understanding. Although the explanatory power of this model was modest, the consistency of attitudinal predictors across both outcomes reinforces the notion that beliefs about the clinical relevance of sustainability are as influential as formal education (18).

Taken together, the regression models reveal that both concern and knowledge are not simply a function of exposure to information, but are deeply shaped by demographic context, disciplinary background, and the perceived legitimacy of sustainability within medical training. These findings provide a strong empirical basis for advocating not only for expanded curricular content, but also for pedagogical approaches that explicitly connect sustainability to professional identity and clinical responsibility.

Attitudes toward curricular content were also highly predictive. Agreement with the integration of sustainability topics in both classroom and clinical settings was associated with greater knowledge and positive attitudes, echoing literature that emphasizes the value of curricular continuity from theoretical to practical learning environments (17, 18). Notably, students who believed that pollution and climate change are relevant to patient care were more engaged, whereas those who viewed these topics as marginal to clinical work were less knowledgeable. This highlights the need to communicate the clinical implications of climate change, such as the rise in noncommunicable diseases, vector-borne illnesses, and mental health effects linked to environmental stressors (19, 20). Experiential learning also emerged as a critical component. Students who expressed a preference for learning sustainability topics through clinical experience had higher odds of both knowledge and positive attitudes. This supports pedagogical models that embed environmental literacy into real-world clinical rotations and interprofessional teamwork (21, 22). The World Health Organization has similarly called for capacity-building approaches that strengthen climate resilience and promote environmentally sustainable healthcare systems (23). In addition to educational variables, perceptions of responsibility were significant. Students who believed healthcare professionals have a duty to use resources responsibly and prevent pollution were more likely to be knowledgeable, underscoring the ethical dimension of sustainability in medicine. This aligns with the Lancet Commission’s call for clinicians to lead climate action by modeling environmentally responsible behavior and advocating for systemic change (24). However, persistent barriers remain. A substantial number of students cited curricular overload and limited training as obstacles to addressing sustainability. These findings echo international reports that medical curricula still lack comprehensive climate-health education, despite growing consensus on its importance (25, 26). Furthermore, misconceptions about single-use medical devices—such as the belief that they are inherently safer—continue to undermine sustainability efforts and suggest the need for clearer evidence-based guidance on safe and sustainable practices (27).

In line with international literature, future educational initiatives should address the specific competencies needed to manage the ecological footprint of healthcare delivery effectively, particularly balancing infection control with environmental sustainability. Studies have emphasized the importance of incorporating life-cycle assessment, waste reduction, and resource optimization into health curricula to enable professionals to make informed decisions regarding the use of single-use versus reusable medical devices (28, 29). Evidence from recent sustainability frameworks suggests that while single-use devices can reduce cross-contamination risks, their widespread adoption significantly increases plastic waste and carbon emissions, whereas properly managed sterilization and reprocessing systems—or hybrid/reusable alternatives—can offer lower overall environmental impacts without compromising patient safety. Therefore, equipping students and clinicians with the knowledge to evaluate these trade-offs represents a crucial step toward environmentally responsible healthcare (30, 31).

## 5 Limitations

This study has several limitations that should be acknowledged. First, its cross-sectional design does not allow for causal inference between awareness, knowledge, and attitudes toward healthcare sustainability. Second, the use of self-administered questionnaires may have introduced self-report and social desirability biases, potentially leading participants to overstate environmentally favorable opinions. Third, as data were collected from a single university, the findings may not be generalizable to all medical and health professional students in other regions or countries. Moreover, this study included several statistical comparisons based on attitudinal and perceptual variables, which may increase the risk of type I error due to multiple testing. Although we used multivariable regression to reduce spurious associations and applied backward selection to limit the number of predictors, no formal multiple-testing correction was applied. Results should therefore be interpreted as exploratory, and confirmatory studies are warranted to validate these associations.

## 6 Conclusion

This study highlights the urgent need to integrate environmental sustainability into medical and health professional education. Both individual and institutional factors influence knowledge and attitudes, and interventions should be multifaceted—targeting curriculum reform, faculty development, and clinical practice environments. Future research should evaluate the impact of longitudinal curricular innovations and explore how sustainability education translates into practice behaviors after graduation.

## Data Availability

The original contributions presented in the study are included in the article/supplementary material, further inquiries can be directed to the corresponding author.
